# The growing disparity between clinical trial complexity and investigator compensation

**Published:** 2010

**Authors:** LJ Burgess, NU Sulzer

**Affiliations:** TREAD Research/Cardiology Unit, Department of Internal Medicine, Tygerberg Hospital and University of Stellenbosch, Parow, South Africa; TREAD Research/Cardiology Unit, Department of Internal Medicine, Tygerberg Hospital and University of Stellenbosch, Parow, South Africa

The issue of investigator compensation in clinical trials is a contentious one, with opinions varying widely between academic researchers, clinical trialists and pharmaceutical companies. Many academic researchers maintain that clinical trial budgets are excessive. Clinical trialists are usually of the opinion that the study budgets are inadequate considering the many potential safety issues, the continuous monitoring of patients and the many ‘hidden costs’ involved in clinical trials. Pharmaceutical companies and clinical research organisations (CROs) are invariably of the opinion that their study budgets are appropriate.

The Tufts Centre for the Study of Drug Development (CSDD) recently conducted a study that examined the impact of protocol design on clinical trial performance. The results indicate that the median number of procedures per clinical trial increased by 49% from 2000–2003 to 2004–2007, while the total effort required to complete these procedures grew by 54%.^1^ According to the author, ‘more complex and burdensome protocols are extending study cycle times, increasing costs and challenging patient recruitment and retention’.^1^

In addition, the rise in the number of eligibility criteria used to screen volunteers has negatively affected the number of volunteers enrolling in clinical trials. This study also found a wide variability in the complexity between therapeutic areas and clinical study phases. Overall, growth in complexity grew at the slowest rate for phase III protocols as companies begin to gather more data in the early phases of clinical research in an attempt to minimise costs.^1^

There have been growing concerns among investigators regarding grant amounts and the slow payment process.^2^ A review of more than 52 000 study contracts from the CSDD showed that grant size has remained relatively constant since 1998, while the number of procedures per protocol has risen drastically.^2^ In addition, the average dollars paid per procedure per patient has declined by 27% over that same period.^2^ This review also reported that it takes pharmaceutical companies on average 140 business days to pay an investigator for work performed.^2^

## Retrospective analysis

We conducted a study to investigate firstly, the average payment per patient per visit at our site, and secondly, to determine the time taken from date of the patient’s visit to date of site payment for CROs and for pharmaceutical companies. The study was conducted by TREAD Research, a site-managed organisation (SMO) based at Tygerberg Hospital, Parow, Western Cape, South Africa. Random clinical trial agreement (CTA) budgets for studies conducted at this site between 2004 and 2009 were retrospectively analysed for the average payment per patient per visit.

An additional analysis retrospectively explored a further 20 randomly chosen studies conducted at this site over the past 10 years (1999–2009). This analysis included 10 studies conducted by CROs and 10 by pharmaceutical companies. The 20 studies included in the analysis all had similar procedures and study designs. The analysis tracked patient visit dates, invoice dates and the date that payment was reflected in the site’s bank account. All data were entered into an excel spreadsheet and descriptive statistics were used to analyse the data.

## Major findings

A total of 33 studies’ budgets were analysed, including 28 outpatient risk-factor studies (14 type 2 diabetes mellitus, 10 hypercholesterolemia and four hypertension) and five cardiovascular (CVS) endpoint studies. These results (as shown in Table 1) indicate an average increase of 13% per patient per visit since 2004 for the risk-factor studies (from R2 215 to R2 550) while the CVS endpoint studies’ budgets decreased by 7% over the same time period (from R3 525 to R3 287).

**Table 1.** Average Payment Per Patient Per Visit Per Year In South African Rand As Well As The Percentage Change In Payment Over The Six-Year Period (2004–2009)

*Study type*	*2004*	*2005*	*2006*	*2007*	*2008*	*2009*	*Percentage change 2004 – 2009 (%)*
Risk factor studies (*n* = 28)	2215	2208	1796	2678	2748	2550	+13%
CVS studies (*n* = 5)	3525	1143	2950	2950	2969	3287	–7%

**Fig. 1.**
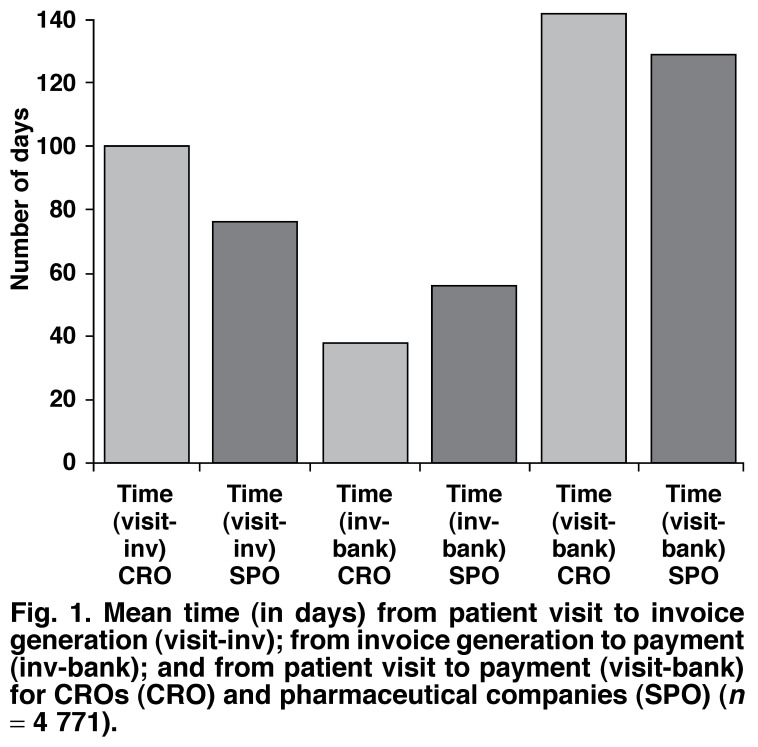
Mean time (in days) from patient visit to invoice generation (visit-inv); from invoice generation to payment (inv-bank); and from patient visit to payment (visit-bank) for CRO s (CRO ) and pharmaceutical companies (SPO) (*n* = 4 771).

An additional analysis investigated the time from the patient’s visit to payment of the receipt, including the time from the patient’s visit to invoice generation, as well as invoice generation to site payment. A total of 4 771 patient visits were included in the analysis, 2 433 (51%) from CROs and 2 338 (49%) from pharmaceutical companies. This resulted in 67 invoices being generated; 34 for CROs and 33 for pharmaceutical companies. The results of this analysis are presented in Fig. 1.. On average, the time from the patient’s visit to site payment was 134.3 days (4.5 months) for CROs and 122.7 days (4.0 months) for pharmaceutical companies.

The main findings of this retrospective analysis are that payments per patient per visit over the past six years at this site have not kept up with local inflation figures and that risk-factor studies’ payments increased by 13% yet the CVS endpoint studies’ payments decreased by 7% over the same period. The average time from patient visit to payment receipt was 134 days (4.5 months) on average for CROs and 122 days (4.0 months) for pharmaceutical companies.

## Billing for ‘hidden costs’

Typically, sites in South Africa only receive the study budget once they have agreed to conduct the study and there is seldom any mention in the contract of annual inflation adjustments, despite the fact that site expenses such as staff-related costs, consumables, equipment and infrastructure-related costs increase annually by at least 8% locally. An unspoken rule of thumb in the clinical trial industry locally has been to charge Medicines Association of South Africa (MASA) rates plus 10%. There is, however, no standardised or formalised approach to billing for procedures or components specific to clinical trials.

Kaiser Permanente is a large American research institute, which recently implemented a system of analysing and billing for the various components of clinical trials. These components include ‘direct components’ such as the costs of specific clinical procedures and research costs versus ‘indirect costs’, such as those incurred by supporting a site’s infrastructure, especially regulatory and administrative costs, which are by their nature ‘hidden costs’.^3^ The equation states that the per-patient ‘direct costs’ plus ‘indirect costs’ equal the total per-patient rate for the study. Their approach translates research and administrative activities into relevant costing codes by using the same logic as that applied to clinical procedures when claiming from medical aids, for example.^3^ Examples of some of these costs inherent to clinical trials include site staff time at investigator meetings, time spent with study monitors, advertising costs, archiving fees and time required to report serious adverse events (SAEs) and endpoints. Other ‘hidden costs’ for which sites are not remunerated include audit preparation costs, unscheduled visits, screen failures and pre-screen costs.^2^

## Increasing clinical trial complexity

Clinical trial budgets have also not taken the median increase in procedures per clinical trial into account. Getz reported a 49% increase in procedures from 2000–2003 to 2004–2007, while the total effort required to complete these procedures grew by 54%.^1^,^2^ This increase in site effort and time is not reflected in the per-patient payment schedule, with a reported 27% decline in per procedure payment over their analysis period. Their study also found a wide variability in the complexity and execution burden between therapeutic areas and clinical study phases.

The present study compared risk-factor and CVS endpoint studies and found that average payment for CVS endpoint studies has decreased by 7% over the last six years at this site. This is despite the fact that these endpoint studies require an incredible amount of manpower to complete the required reporting needed to meet their primary statistical endpoints. The American Food and Drug Administration (FDA) drafted a recommendation: ‘Endpoints and standardised data collection for cardiovascular endpoint trials’ in July 2009. These recommendations, if and when adopted, will mean that site staff will be spending even more time collating and submitting data for these CVS endpoint trials.

## Delayed payment

Our study also found that the time from patient visit to site payment took on average 128.5 days (mean time in days from both the CROs and pharmaceutical companies). The study by Getz^2^ reported that it takes pharmaceutical companies on average 140 business days to pay an investigator for work already performed. The present study had similar findings, with CROs taking on average 134 days to pay and pharmaceutical companies taking 122 days. This delay in payment is often despite agreed payment deadlines in the CTA. Such payment delays have hugely adverse effects on a site’s cash flow since monthly expenses and creditors still need to be paid timeously.

While there may be many contributing factors to explain this excessive payment delay, one reason often cited by pharmaceutical companies and CROs alike is that they are unable to pay for work that has not been monitored. Invariably, in rapidly recruiting studies and studies that generate a large volume of data, the monitoring visits fall behind and therefore the time to payment lengthens. A possible solution would be to ensure that allowances be made in study budgets for more frequent monitoring visits, especially during the recruitment phase of the study. This would ensure that monitoring remains on track with data collection, invoices can be generated faster, and payments processed within a more reasonable time frame.

## Conclusion

Investigators and the pharmaceutical industry alike need to acknowledge that clinical research has to be run like a business.2 This includes making allowances for annual inflation in study budgets, incorporating all trial-related procedures into the budget and taking pro-active measures to ensure that site payment is made promptly.
